# What is known from the existing literature about adolescent knowledge and attitudes towards dementia and interventions to enhance this? A scoping review

**DOI:** 10.1371/journal.pone.0322423

**Published:** 2025-09-08

**Authors:** Niamh McCarthy, Svea Nagel, Gabriel Vaughan, Emma Berry, Gillian Carter, Lisa Graham-Wisener

**Affiliations:** 1 School of Psychology, Queens University Belfast, Belfast, Northern Ireland, United Kingdom; 2 School of Nursing and Midwifery, Queens University Belfast, Belfast, Northern Ireland, United Kingdom; Ladoke Akintola University of Technology Teaching Hospital: LAUTECH Teaching Hospital, NIGERIA

## Abstract

**Objectives:**

Individuals with dementia often experience stigmatisation and social exclusion. To develop dementia-friendly communities, it is important to take a life-course approach to enhance the perception of dementia in the general population. Currently, we lack an overall understanding of adolescent perceptions of dementia. This scoping review is the first to identify the existing literature on the knowledge and attitudes held by adolescents (aged 10–19 years) towards dementia.

**Methods:**

A JBI Scoping Review. Four bibliographic databases (MEDLINE, PsycINFO, EMBASE, and CINAHL) were searched to identify eligible studies. Eligibility criteria included qualitative and quantitative studies examining the knowledge and attitudes of adolescents (10–19-year-olds) towards dementia. Studies were screened for eligibility, with data extracted using JBI tools and synthesised in relation to i) knowledge and attitudes and ii) interventions.

**Results:**

In total 21 publications were identified from the UK, Slovenia, the United States, Taiwan, Canada, Israel, South Africa, and South Korea. Ten of these studies included interventions. Overall, studies reported adolescents to have a relatively low level of objective knowledge about dementia. Conversely, attitudes towards dementia were largely reported to be neutral-positive. However, adolescents living with a relative with dementia often held predominantly negative attitudes towards the condition. Gender and having a family member living with dementia were important predictors of both knowledge and attitudes. Interventions to date are educational or intergenerational in focus and appear similarly effective in increasing attitudes and awareness towards dementia.

**Conclusions:**

The current scoping review identified a small but emergent body of literature on adolescent knowledge and attitudes towards dementia. The synthesised findings indicate low objective knowledge to be a particularly useful target for intervention and alongside other findings, will be useful to inform future research, particularly the development of high-quality intervention studies.

## Introduction

The aging of societies worldwide has shifted focus to aging-related impairments such as dementia, which has emerged as a global health challenge [1–2]. Dementia has a significant impact on national economies, but most crucially, on individuals and their families [3]. The epidemic growth of dementia was acknowledged by the World Health Organisation (WHO) [4] as a public health priority, with estimates that there will be 139 million people diagnosed worldwide with dementia by 2050. Dementia is defined in this scoping review as a syndrome comprising a large number of differing brain disorders which are usually of progressive or chronic nature [5]. Alzheimer’s Disease is considered the most common type of dementia worldwide [6].

A lack of dementia education and awareness often results in the stigmatisation of people living with dementia and barriers to diagnosis and care [5]. Stigma is an attribute that is profoundly discrediting in a social interaction [7] and which may lead to separation, stereotyping, labelling, and discrimination [8]. Stigma in the context of dementia is often due to a lack of awareness and fear of the unknown [9]. People living with dementia and people supporting someone with dementia can be significantly affected by stigmatisations, with consequences such as isolation, low self-esteem, poor mental health, and reduced quality of life [10].

While there has been promising evidence of an intervention beneficial in both dementia-stigma reduction and improving dementia knowledge, [7] there remains a lack in abundance of effective approaches to minimise dementia-related stigma [7], despite stigma having been established as one of the major concerns for people living with dementia and their carers [11]. Although dementia-related attitudes and knowledge have seen an increase in research attention over the past decade, targeted groups were mostly health care professionals and/or students [12,13]. There is a lack of evidence exploring the general population’s knowledge and attitudes toward dementia, and particularly among younger populations such as adolescents [14].

Adolescence is defined in this scoping review as a person between the ages of 10 and 19 years [15]. Adolescence is a formative period in which significant changes in a person’s cognitive, biological, social, and psychological characteristics occur and many new life patterns are learned and established [16]. Adolescent knowledge and attitudes towards dementia and how they form is important, considering evidence that stigma towards mental health develops at a young age and is maintained across a lifespan [17]. Therefore, adolescence represents a key opportunity for intervention towards avoiding lifelong stigmatisation of the disease and developing dementia-friendly communities [18]. Furthermore, considering the epidemic growth of dementia, the probability for the younger generation to encounter people living with dementia, or become carers themselves, is increasing [19]. A survey conducted by the Alzheimer’s Society [20] highlighted the importance of educating young people, as two thirds of the young people who responded reported that their lack of knowledge about the disease prevented them from helping people living with dementia. This underpins the importance of considering how to equip adolescents with the ‘tools’ (e.g., knowledge/skills) to communicate with/support people with dementia.

In their global action plan on the public health response to dementia, the WHO [21] recommended increasing awareness of dementia to ensure that society is inclusive of people living with dementia and those who care for individuals with dementia. Therefore, it is vital that we gain a comprehensive understanding of the existing research literature to inform research and practice. This scoping review aims to identify from the existing literature, what is currently known about knowledge and attitudes towards dementia by adolescents (10–19-year-olds), and how this can be enhanced. A preliminary search of the Cochrane Database of Systematic Reviews, Prospero, Open Science Framework, and JBI Evidence Synthesis was conducted and no current or underway systematic reviews or scoping reviews on the topic were identified.

**Objectives:**

1. To examine the knowledge and attitudes adolescents have about dementia.
2. To explore key stakeholder (parent, guardian, grandparent, teacher and adolescent) perceptions of how adolescent knowledge and attitudes towards dementia can be enhanced.
3. To review the intervention strategies and/or designs that have been used to enhance adolescents’ knowledge and attitudes towards dementia and their effectiveness.

**Research question**:

What is currently known about adolescent knowledge and attitudes towards dementia and how this can be enhanced?

## Methods

### Design

This scoping review followed the Joanna Briggs Institute (JBI) methodology. The JBI approach to scoping reviews offers a systematic and internationally recognised method to map the extent, range and nature of evidence on a topic [22]. It emphasises clear objectives, inclusion criteria and transparent reporting following the JBI Manual and PRISMA-ScR guidelines [22–23].Conducting a scoping review is beneficial as it allows an analysis of the overview of a research area and identifies gaps in the research which can further inform future research studies [24]. A scoping review was therefore found to be an appropriate method, as evidence around the topic of adolescents’ knowledge and attitudes towards dementia is relatively new [24] and consequently the evidence for knowledge and attitude enhancing interventions are also new and have not been systematically identified and explored [25].

The scoping review protocol was registered with the Open Science Framework (https://osf.io/n4hua/) and the review is being outlined in accordance with the Preferred Reporting Items for Systematic Reviews and Meta-Analyses Extension for Scoping Reviews [26] (S1 Appendix).

### Population

This review included studies of adolescents aged between 10–19 years, according to the WHO [15] definition.

The following definitions were included: teenagers, young adults, youths, young people/young person, and juveniles within the stated age range [27–28]. Participants included adolescents who do not have any direct contact with dementia, e.g., who do not have relatives and/or friends living with dementia and adolescents who do have family and/or friends who live with dementia.

Exclusion criteria included children under the age of 10 years, young adults, and university students over the age of 19 years if specified in the studies. This review included studies irrespective of the type of dementia. These included Alzheimer’s disease, mild and severe dementia, early dementia, Lewy body, mixed, frontotemporal, and vascular dementia.

Studies were excluded if the targeted population’s data could not be extracted separately. Older adult participants with a mixed sample of neurodegenerative diseases as well as dementia were not included. Studies which involved mild cognitive impairment (MCI) were excluded due to the more significant disruptions to daily activities and social functioning which dementia poses in comparison to MCI [29]. There were no restrictions on ethnicity or gender.

### Concept

The concept of this study is to examine the knowledge and attitudes adolescents hold about dementia, as well as the strategies and/or designs of interventions which have been developed to target adolescent knowledge and attitudes towards dementia. Observational and qualitative research designs and interventions examining the knowledge and attitudes of adolescents towards dementia were included.

### Context

This scoping review considered studies from all geographical locations conducted across all settings (e.g., schools and community-based settings) which involve adolescents and their knowledge and/or attitudes towards dementia.

### Types of sources

This scoping review considered both quantitative and qualitative primary research. Including both research methods provides a comprehensive understanding of both measurable data and experiential factors [30]. Furthermore, examining both allows to capture different perspectives and identify gaps in research [30]. This included observational study designs (e.g., descriptive or analytical cross-sectional studies), as well as experimental and quasi-experimental study designs (e.g., randomized controlled trials, before and after studies). Qualitative designs considered included, but were not limited to, designs such as phenomenology, grounded theory, ethnography, qualitative description, action research, and feminist research.

Studies published in English only were included due to limited resources within the research team to support translation. Studies published between 2000–2024 were included as they hold the most current knowledge and recent intervention practices that are most relevant to present adolescent perceptions.

Systematic reviews were excluded but were considered for relevant papers found in their reference sections. Grey literature were excluded to ensure greater consistency and quality in the evidence-base.

### Search strategy

Exploratory searches of MEDLINE, PsycINFO, EMBASE, and CINAHL were undertaken to identify articles on the topic. This was followed by an analysis of the index terms used to describe the article, as well as the compositions of the words in the title and abstract. A formal search strategy was constructed for each bibliographic database around three key concepts: dementia, adolescents and knowledge and attitudes included as one key concept as key words related to each (See S2 Appendix for search terms).

A formal search was conducted to identify studies published in English between 1^st^ January 2000 and 15^th^ of April 2024. Following the search, all studies were exported to EndNote 20.2 [31]. After removing duplicates, studies were transferred from EndNote to Rayyan [32], delegated equally amongst three reviewers and abstracts were each screened independently by two of three reviewers (N.MC., S.N. & G.V.) against the eligibility criteria. Changes or conflicts were solved through discussion and consensus within the research team. This process was repeated with the full text of studies, with included articles delegated equally amongst three reviewers and independently screened by two of three reviewers (N.MC., S.N. & G.V.). The references and citation lists of the included studies were then reviewed.

### Data extraction

An adapted version of the JBI Data extraction form [33] was used to extract and chart the data from included studies (See S3 Appendix), following a pilot test and refinement by authors. Data was extracted by each of the three researchers (N.MC., S.N. & G.V.) and was scrutinized for accuracy by one of the three researchers independently (N.MC., S.N. & G.V.). The extracted data included details about the author(s), year, setting, sample size, participants, study design(s), intervention(s), aim(s), finding(s), recommendation(s), and limitations relevant to the research question. Disagreements were resolved through discussion and/or with an additional independent reviewer.

### Data synthesis and presentation

Data from the extraction was presented in an overall data summary table. A narrative summary of the study characteristics and findings was then developed, aligned to each study objective, with tabulated results. The TIDIER framework was used to review interventions. This framework ensures interventions are described in sufficient detail to ensure accurate interpretation and replication for future research [34]. For example, one of the components of the framework is how the intervention is delivered – this may be in person, online or by telephone conducted in groups or individually [34]. Discussion of gaps and overlaps in the literature were then discussed including further areas of research and proposals of the best intervention practices.

## Results

### Study selection

**Fig 1:**
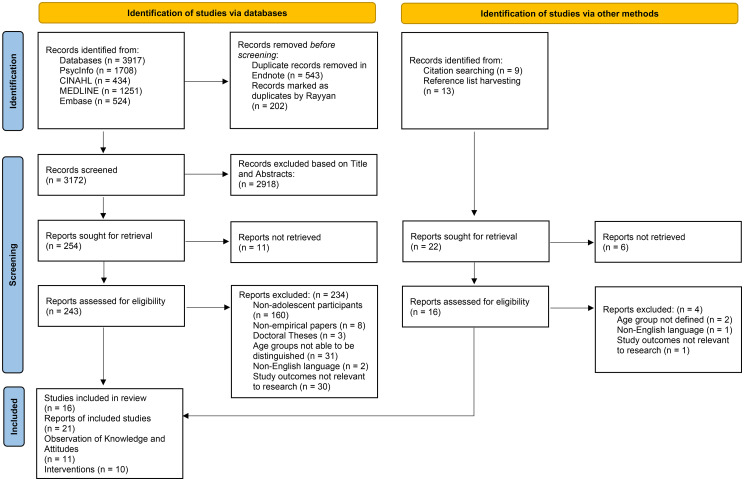
PRISMA-ScR flow diagram showing the article selection process.

The searches of the electronic databases resulted in 3917 articles in total. A total of 2918 articles were excluded based on the title and abstract, which resulted in 243 articles being retrieved and assessed in full for eligibility. After full-text review, the number of articles was reduced to nine. Through reference list and citation harvesting, 16 potentially eligible articles were full text screened from which 12 were retained. Therefore, a total of 21 articles (Table 1) were included in this scoping review. The search strategy is shown in the PRISMA-ScR flow diagram (Fig 1).

**Table 1:** Data summary: Studies included in the review.

Authors	Participants	Aim	Design	Data collection	Intervention	Control	Outcome	Main findings
Alant et al. (2015) South Africa	7 students in a high school 15-16 years Gender not reported	Exposing participants to older persons with dementia using the Memory Bridge Initiative (MBI) to develop empathetic skills.	Qualitative thematic analysis	Pre-post focus group discussions	3.5-day Memory Bridge Initiative (school programme with people with dementia)	No	NA	Students had a positive perception of ‘older person’ and ‘person with Alzheimer’s disease’ post MBI. Frequency of positive associations for ‘person with Alzheimer’s’ increased from two to nine post MBI and negative associations reduced from 15 to nine. All participants described older person in positive language post MBI. Dementia knowledge was not measured in this study.
Chow et al. (2018) Canada	4 adolescents in a high school 15-17years Gender not reported	Increase the community’s knowledge and appreciation of dementia, reducing the stigma surrounding the illness, and providing the opportunity for students to volunteer with people with dementia.	Quasi- experimental	Pre-post measures Thematic analysis of the debriefing rounds.	Student visited the care home on a weekly basis where each student was paired with a resident with common interests.	No	Palmore’s ‘’Facts on ageing quiz” (Knowledge). A survey specifically developed for this program. (Attitudes).	The thematic analyses found that students learned a lot about both themselves and dementia. Students expressed significantly more positive attitudes towards dementia. Descriptive statistics not reported.
Farina et al. (2020) England	901 adolescents in a high school 13-18 years 423 Males 478 Females	Explore the perceptions and experiences of dementia in an adolescent sample.	Cross-sectional observational	Quantitative survey design	NA	NA	Adolescent Attitudes towards Dementia Scale (Attitudes).	Adolescents had positive/neutral attitudes. A small proportion (28.5%) held negative attitudes. 287 adolescents responded ‘yes’ to wanting to learn more about dementia (32.0%), which is a similar number to those who responded ‘no’ (n = 321, 35.7%) or ‘maybe’ (n = 290, 32.3%).
Farina et al.(2020a) England	301 adolescents in a high school 12-16 years 129 Males 172 Females	Understand the short-term effect of the Dementia Friends initiative on adolescents’ attitudes towards dementia whilst gaining some insight into their satisfaction of the session.	Quasi- experimental	Pre-post measures	Classes were assigned to receive Dementia Friends (dementia awareness group)	Lessons as usual	The Brief Adolescent Attitudes towards Dementia Scale (A-ADS) (Attitudes). The Kids Insight into Dementia Survey (KIDS). (Perceptions)	Dementia Attitudes: The average KIDS score (t =−5.57, p < 0.0001) significantly improved between pre and post for the dementia awareness group. The Brief A-ADS reported no statistically significant change between time points (t = 0.29, p = 0.77). When investigating change scores between groups, there was no statistically significant difference in attitudes and perception between the dementia awareness group and control group on the KIDS and Brief A-ADS outcomes. Effect sizes ranged from non-existent on the KIDS (d=−0.003, p = 0.98) to small on the A-ADS (d = 0.14, p = 0.31). Dementia knowledge was not measured in this study.
Farina et al. (2020b) England	30 adolescents in a high school 11-16 years Gender not reported	1.How does Dementia Friends training affect adolescents’ attitudes and knowledge of dementia? 2.What do adolescents think about Dementia Friends sessions and their content?	Qualitative thematic analysis	Focus group discussions	Classes were assigned to receive Dementia Friends (dementia awareness group)	No	NA	Four themes were identified: 1) perceptions and experiences of dementia: Participants had an overall positive perception of dementia and a positive outlook of people. 2) outcomes and learning from Dementia Friends session: participants did not fully understand what dementia was before the session or how dementia affects individuals. 3) reactions to the Dementia Friends session: there was a great deal of positivity about the Dementia Friends. 4) identified future learning needs: Dementia Friends improved their attitudes and knowledge.
Felc & Felc (2021a) Slovenia	1128 adolescents in a high school 14-19 years 30.9% Males 68.3% Females	To evaluate dementia-related knowledge with a focus on comparison between adolescents with and those without relatives with dementia.	Cross-sectional observational study	Quantitative survey design	NA	NA	Author’s own survey – 20-items on dementia-related facts Students preferred methods of dementia knowledge acquisition.	Out of 20 questions on dementia knowledge, participants answered more than 14 correctly. The higher level of knowledge was statistically significant for students with a relative with dementia (M = 14.67), girls (M = 14.63), students in secondary technical and vocational school (M = 14.73) and in general secondary schools (M = 14.64). Respondents without a relative with dementia showed poorer knowledge of the facts that dementia manifests itself as loss of orientation, speech, recognition, inappropriate behaviour, that the number of people with dementia increases after age 65, that the most common cause of dementia is Alzheimer’s disease, and in the belief that a person with dementia can live at home (p < 0.05). A subsample of 335 adolescents (55%) with a relative of dementia showed sadness and fear in their attitudes towards dementia.
Felc & Felc (2021b) Slovenia	Same as Felc & Felc (2021a)	To determine whether gender differences in knowledge of dementia exist and desired resources for further education.	Same as Felc & Felc (2021a)	Same as Felc & Felc (2021a)	NA	NA	Same as Felc & Felc (2021a).	Results of the first and third sections of the questionnaire are the same as Felc & Felc (2021a). Student suggestions for further dementia education did not differ between girls and boys (p > 0.05). The comparison of gender proportions did not show statistically significant differences in the results (p > 0.05). More than two-thirds (69.4%) want to get information about dementia online, a fifth (22.7%) from health professionals and a fifth (20.3%) from books, textbooks, magazines, newspapers and information points.
Felc & Felc (2021c) Slovenia	Same as Felc & Felc (2021a)	To assess dementia-related knowledge with a focus on comparison between female and male adolescents and whether any stereotype about older adults is expressed among them.	Same as Felc & Felc (2021a)	Same as Felc & Felc (2021a)	NA	NA	Same as Felc & Felc (2021a).	Results of the first and third sections of the questionnaire are the same as Felc & Felc (2021a). Analysis of additional item- impaired memory is a normal part of getting old. As many as 86.0% of girls and 81.9% of boys agreed. There was no statistically significant difference in gender for this statement.
Felc et al. (2021) Slovenia	Same as Felc & Felc (2021a)	To evaluate knowledge of dementia and attitudes towards people with dementia among Slovenian non-health related students.	Same as Felc & Felc (2021a)	Same as Felc & Felc (2021a)	NA	NA	Same as Felc & Felc (2021a).	Sadness and fear are common in the attitudes of adolescents with a relative with dementia (55% of all responses). Only 11.3% of responses expressed an optimistic attitude. A positive attitude was also present in an additional 12.5% of responses, who believe that experiencing changes in a relative due to dementia is easier and less painful when they are educated about dementia.
Felc (2022a) Slovenia	Same as Felc & Felc (2021a)	To determine the knowledge of dementia among students and to identify their suggestions to acquire dementia-related knowledge.	Same as Felc & Felc (2021a)	Same as Felc & Felc (2021a)	NA	NA	Same as Felc & Felc (2021a).	Both, girls and boys in the same percentage indicated future priority learning methods namely: use of the internet, learning in school lessons, from books and textbooks and learning from medical staff.
Felc (2022b) Slovenia	Same as Felc & Felc (2021a).	To evaluate adolescents’ knowledge of dementia and attitudes towards behavioural and psychological changes in persons with dementia.	Same as Felc & Felc (2021a).	Same as Felc & Felc (2021a).	NA	NA	Same as Felc & Felc (2021a).	Sadness and fear are common in attitudes towards dementia in almost two thirds (64.5% of all responses) of the respondents with a relative with dementia. 13.6% of responses expressed positive attitude towards people with dementia. Positive attitude was reflected in 15.1% of the responses. 19.4% of responses indicated that they have neutral attitudes towards dementia.
Fuh et al. (2005) Taiwan	5825 adolescents in high schools 10-15 years 2985 Males 2840 Females	Assess the attitude towards dementia among children and adolescents. Investigate the relationship between selected demographic characteristics and these attitudes.	Cross-sectional observational study	Quantitative survey design	NA	NA	Attitude Toward Dementia Questionnaire. (Attitudes).	The older students and girls had a significantly higher frequency of having heard of dementia; 95.0% girls vs. 91.8% boys, (p < .001). Personal knowledge of a person with senile dementia was not related to age and gender (p > 0.05). For attitudes, boys and younger students felt embarrassed to invite classmates’ home if they had a relative with dementia; boys 8.5% vs. girls 5.3%, (p < .001).
Isaac et al. (2017) United Kingdom	358 adolescents in a high school 15-18 years 127 Males 231 Females	Improving people’s knowledge, perceptions and attitudes of dementia is important in the formation of dementia-friendly communities. To evaluate adolescents’ knowledge and attitudes of dementia.	Cross-sectional observational study	Quantitative survey design	NA	NA	Author’s own survey (Knowledge & Attitudes)	Out of 15 questions on dementia knowledge, participants were on average able to answer less than half correctly [M = 6.65, standard deviation (SD) = 2.34]. Neither year of study, school, nor gender, affected scores on the dementia knowledge questionnaire (p > 0.05). Adolescents that knew someone with dementia scored significantly higher on the knowledge questionnaire (M = 7.15, SD = 2.23) compared to those that did not know anyone with dementia (M = 6.52, SD = 2.34) (U = 9718.00; p* = *0.03). Attitudes were mixed. They were positive to statements which looked at those with dementia as an individual: ‘People with dementia should be involved in activities in the community’ (Mdn = 2.0, IQR = 1.0) and negative attitudes towards caring for them: ‘There comes a time when all you can do for someone with dementia is to keep them clean healthy and safe’ (Mdn = 2.0, IQR = 0.0).
Liao et al. (2022) Taiwan	200 adolescents in high school 12-18 years 152 Females 48 Males	To compare the long-term effects of exergaming (Kinect) and companionship programs on attitudes towards dementia and older adults among adolescents.	Quasi-experimental	Pre-post measures	The adolescents were assigned to five groups: 5-week exergaming, 5-week companionship, 8-week exergaming, 8-week companionship.	School activities instead of intergenerational activities.	Dementia Attitudes Scale (Attitudes). The Attitudes Toward Elderly modified scale (Attitudes).	Attitudes from interacting with people with dementia in the 8-week exergaming group had a significantly better attitude than the control group post-test and at 1,3 and the 6-month follow-up. Results of the 8-week companion group also showed a significantly improved attitude compared with the control group post-test and at the 6month follow-up. Furthermore, the 5week companion group showed a significant improvement compared with the control group only at the post-test. The 5-week exergaming group showed no differences in attitudes post-test or at 1, 3- and 6-month follow-up with the control group. Dementia knowledge was not measured in this study.
McNair & Moore (2010) USA	17 adolescents in a high school 13-15 years 11 Males 6 Females	To explore the effects of intergenerational programs on individuals with Alzheimer’s disease or dementia and the younger generations’ attitudes and perceptions of older adults.	Quasi-experimental	Pre-post measures	One to two students were matched to each elder through common activity interests and personality traits. Six one-hour programs were developed and were conducted once per week.	No	Children’s Views on Aging (Attitudes). Two-open ended questions.	Despite the finding of no significant changes when comparing the pre and post CVOA attitudinal survey, the adolescents stated that their comfort level with the elders with dementia changed. Adolescents were asked how they felt pre and post intervention. 82% felt nervous and/or scared prior to meeting their senior friends and 71% of adolescents stated that they “felt good” after spending 8 weeks with the senior friend. Dementia knowledge was not measured in this study.
Parveen et al. (2015) England	38 adolescents at high schools 14-17 years Gender not reported	To foster a positive attitude towards those living with dementia and to encourage a friendly community.	Quasi-experimental	Pre-post measures	Educational Programme Workshop	No	Authors developed a 10-point Likert scale (Knowledge).	Knowledge improved from an average score of 4.5 to 8 out of 10. After the intervention the average score improved to 7.8. Attitudes towards dementia were not measured in this study.
Parveen et al. (2020) United Kingdom	42 adolescents at high school 12-18 years 10 Males 32 Females	To explore the dementia related educational needs of adolescents.	Qualitative, thematic analysis	Focus groups	NA	NA	NA	Adolescents expressed an interest in learning more about dementia and perceived awareness of dementia to be important within their age group. A significant number of the students had some personal experience of dementia and wished they had been more prepared. Most students expressed a desire to learn more about the risk factors associated with dementia and how to reduce one’s own risk.
Saif et al. (2021) USA	172 adolescents in high school Mean age 16 years 61 Males 111 Females	To evaluate the effectiveness of various online education strategies concerning Alzheimer’s Disease (AD) risk reduction and brain health in younger populations.	Randomised controlled trial	Pre-post measures	4 different educational interventions.	Reduced interaction noncelebrity/nondoctor narrated video lesson.	Authors own pre- and post-survey to collect knowledge/beliefs regarding AD. (Knowledge).	Mean pre-lesson scores were 4.9 (SD 2.9) of 9 for high school students; these did not differ among the 4 groups in high school students (p = 0.54). In high school students, the celebrity webinar, celebrity video, and doctor webinar had greater improvements in awareness that, both nutrition and exercise may reduce AD risk compared to the control group. All 4 groups scored higher on the post-lesson knowledge quizzes and increased post-lesson. Attitudes towards dementia were not measured in this study.
Song et al. (2019) South Korea	32 adolescents in high school 17-19 years 21 Males 11 Females	This study seeks to find out what kind of influence does the Dementia Education Program (DEP) have on adolescents.	Quasi-experimental	Pre-post measures	Participant’s received DEP consisted out of 9 kinds of education subjects for 4 hours a day, 2 days in total.	No	Dementia awareness questionnaire (Dementia awareness).	Before DEP, the score was 10.15 ± 1.52. After DEP, the score was 12.37 ± 1.75. Comparing before and after scores, the score increased. The result showed statistical significance (r < 0.01). The effect of awareness towards dementia on the DEP from adolescents was positive.
Werner et al. (2017) Israel	460 adolescents in high school 14-15 years 55.1% female 44.9% male	To assess stigmatic beliefs toward a person with Alzheimer’s Disease (AD) in high-school students and to examine whether majority minority status is associated with stigmatic beliefs beyond other correlates of stigma.	Cross-sectional observational study	Quantitative survey design	NA	NA	Alzheimer’s Disease Knowledge Scale (Knowledge).	Across all the dimensions, stigmatic beliefs toward a person with AD were below the neutral score of 4.5, reflecting low levels of stigma. Slightly higher levels of ageist beliefs were reported in two of the three dimensions of ageism contribution and stereotype. Knowledge levels were also moderate, with 52.3% answering correctly to knowledge items. Approximately 69.5% knew someone with AD.
Wescott & Healy (2011) USA	15 adolescents in high school 16-18 years 8 females 7 males	To examine high school students’ experience of participating in an adaptation of the Memory Bridge Initiative (MBI) in Maine.	Quasi-experimental	Pre-post measures	Educational MBI programme involving students paired with a dementia ‘buddy’ for four weeks.	No	Authors own pre-and post-questionnaire to assess the impact of the programme on students’ perceptions of Alzheimer’s Disease (AD).	Quantitative: Students’ positive words of AD increased by 13%, and their negative words decreased by 19.8% post intervention. There was a significant change in students’ perceptions of AD based on negative ranks with 15.5 positive changes relative to no negative changes (z = −5.477, p < .001). Qualitative: Two themes; People first: older people with AD are “still people” and Patience and Empathy in communication “requires understanding”.

### Study characteristics

Of the 21 included reports, 11 explored the knowledge and attitudes of adolescents towards dementia and ten investigated the potential of interventions to increase adolescents’ knowledge of and attitudes toward dementia. The vast majority were published in the United Kingdom (n = 6) and in Slovenia (n = 6), followed by Taiwan (n = 2), and the United States of America (n = 3). One study was published in Canada, Israel, South Africa, and South Korea, respectively. The majority of studies were completed in high income countries with one study in a low-to-middle income country [35].

Four studies exploring knowledge and attitudes of adolescents towards dementia used a cross-sectional design and questionnaires or questionnaire interviews, that were carried out in person or online [14,36–38]. One of these studies [39] included a further five reports which used the same sample but performed separate analyses for different demographics [40–44]. One qualitative study used focus group discussions [45].

Of the studies exploring interventions, seven studies used a quasi-experimental design involving surveys and for some, an embedded qualitative component [46–52]. There was one randomised controlled trial identified [53], and two qualitative studies using focus groups [54–55].

There was a considerable variation in sample sizes between studies, with the smallest study including only 4 adolescents [46], and the largest study comprising of 5825 adolescents [36]. All studies recruited high school students as their adolescent participants.

### Knowledge and attitudes adolescents hold about dementia

This scoping review identified six studies and five additional reports that investigated adolescents’ objective knowledge about dementia [14,36–45]. Two studies found that the participants were able to answer less than half of the dementia knowledge questionnaires correctly [37–38]. However, only one of these studies [38] used a validated measure to assess adolescent’s knowledge (*Alzheimer’s Disease Knowledge Scale*). Fuh et al.’s [36] study found that overall objective knowledge of dementia was at a low standard. From the thematic analysis conducted in Parveen et al.’s [45] qualitative study, adolescents reported that they had limited knowledge of dementia.

The results of the Felc and authors study [39] which produced a further 5 reports [40–44] initially reported more positive findings, with 71.5% of adolescents able to answer correctly in a dementia knowledge questionnaire (author’s own). However, a more in-depth investigation of the items within the survey suggests significant knowledge gaps. For example, 58% of adolescents did not know that dementia can appear as inappropriate behaviour; 85% believed that it is normal to lose memory function with aging; and 77% of adolescents did not know that dementia can lead to loss of speech [39–44].

This scoping review identified three studies and two additional reports which suggested sex, family members with dementia, an urban school, and older age as predictors of better dementia knowledge [14,36,39,41,42]. There was a positive correlation of age and knowledge of dementia with older aged adolescents showing higher levels of knowledge, but this correlation was weak [42]. Those in general secondary schools scored higher on dementia knowledge compared to those in lower vocational schools [42].

Farina et al. [14] found that female adolescents demonstrated more knowledge and a better attitude towards dementia on nearly every item of the Adolescent Attitudes towards Dementia Scale (A-ADS) [56] and the adapted level of contact questionnaire for mental illness [57]. In Felc and colleague’s study [39], when presented with 20 statements about dementia, it was found that males knew significantly less about dementia than females. This was found especially when asked about the definition of dementia, and also the most common form of dementia i.e. Alzheimer’s Disease [39,41,42]. Additionally, males showed significantly less knowledge about risk factors of dementia, where they underestimated possible modifiable risk factors such as smoking, alcohol abuse, and internet addiction [42]. Fuh et al. [36] came to a similar conclusion and reported that young female adolescents were more likely to have heard about senile dementia than males and were less likely to hold the false believe that senile dementia is contagious. Contrastingly, Isaac et al. [37] explored the knowledge and attitudes of adolescents and they found adolescents’ ability to correctly answer questions about dementia was low and found no effect of school, age, or gender on knowledge.

Two studies found that adolescents with a relative or who knew someone with dementia had a better understanding of the disease. Felc [41] found that those with a relative with dementia scored higher on questions such as if dementia causes cognitive decline, if diagnoses increase with age, if Alzheimer’s is the most common dementia and if those with dementia can live at home. Fuh et al. [36] found that when questioned about their knowledge of dementia, adolescents with relatives with dementia still showed misconceptions. Around 69% of adolescents believed that senile dementia is curable; 88% thought that senile dementia is preventable; and 33% of adolescents believed that senile dementia is a normal part of the aging process. However, Fuh et al. [36] highlighted that the questionnaire used in the study focused predominantly on general impression about dementia and was less inclined to test dementia knowledge.

This scoping review identified five studies that investigated adolescents’ attitudes towards dementia [14,37,38,40,54]. Isaac et al. [37] reported mixed attitudes towards dementia with predominantly more negative attitudes towards the care for individuals with dementia. They reported 47.1% of adolescents were of the opinion that people with dementia should be in a residential home, and 59.5% of adolescents agreed that as the dementia progresses, there will be a time in which keeping the person healthy, clean, and safe is all that can be done. Felc [40] reported only 13.6% of adolescents expressed positive attitudes towards people with dementia.

This scoping review identified three studies in which the adolescents expressed a mostly positive/neutral attitude towards dementia [14,38,54]. Two studies by Farina et al. [14,54] identified that adolescents had a positive perception of dementia and in general a positive attitude towards people living with dementia. Around 80% of adolescents reported feeling positive towards dementia; 71% agreed with the statement that they would be interested in learning about the dementia experience from those affected; and the majority of adolescents agreed with the statement that they would be inclined to help individuals with dementia if they noticed them struggling [14]. In an investigation of stigma towards Alzheimer’s Disease among adolescents, Werner et al. [38] similarly concluded that stigma was low on all three dimensions (Cognitive, Behavioural, Emotional).

This scoping review identified two studies which suggested sex and family members with dementia, acted as a predictors of dementia attitudes [14,40]. The cross-sectional study by Felc [40] suggested that the negative attitudes adolescents hold towards dementia is likely rooted in a lack of dementia education and possibly caused by a relative living with dementia. More than half of the adolescents (64.5%) who had a relative with dementia held negative attitudes towards psychological and/or behavioural changes in that relative [40]. Additionally, Farina et al. [14] found that females held a better attitude towards dementia than males on nearly every item of the ‘Adolescent Attitudes towards Dementia Scale’ [14].

### Key stakeholder perceptions on how adolescents’ knowledge and attitudes towards dementia can be enhanced

Adolescent stakeholders from two studies and 5 additional reports noted that adolescents wanted more information about dementia [39–45]. Felc and accompanying authors [39–44] found male and female adolescents were equally interested in learning more about dementia and that most preferred sources were internet websites (70%). Only one-third of the adolescents expressed an interest in learning about dementia from textbooks and teachers within the school setting. Even less, one-fifth of adolescents expressed an interest in learning about dementia from medical staff, such as nurses and physicians, who can educate from first hand experiences, e.g., misconceptions, how to relate to someone with dementia, and the importance of an early diagnosis [41]. It was acknowledged that most dementia knowledge is acquired by adolescents through their family members, with little information provided by teachers and television [42].

Parveen et al. [45] found that adolescents in their study would prefer interactive learning over traditional school lessons. Adolescents recognised that some large-group teaching would be necessary to convey facts and figures but felt small-group activity would be more conducive to not feeling judgement over lack of knowledge. Adolescents suggested using video clips to assist in delivering key messages on the disease to advance their learning.

None of the studies that measured attitudes assessed stakeholder perceptions of how to improve attitudes towards dementia [14,36,37,40]. Additionally, no study measured how either knowledge or attitudes of adolescents can be enhanced from adult perceptions.

### Interventions

This scoping review identified ten interventions that aimed to improve the level of knowledge and attitudes about dementia amongst adolescents. To review interventions, the TIDIER framework was applied to accurately report the intervention components (Table 2) [34].

**Table 2:** Intervention characteristics.

Authors	Intervention type	Rationale/Intervention Goals	Intervention description	Who	How	Where	When and how much	Tailoring
Alant et al. (2015) South Africa	Intergenerational programme	To develop participant’s empathetic skills through interactions with people with irreversible dementia.	Participants paired with an adult with irreversible dementia in a buddy partnership. Participants make an ‘I-Land map’ of their inner world, e.g., memories, people in their life, their fears etc. Lastly, being part of a peer circle to share and communicate.	Memory Bridge Initiative (MBI) Researchers.	Pre and post focus group discussions and meet with older person twice for 45–60 minutes.	School	Three and a half days. Total of 20 hours.	None.
Chow et al. (2018) Canada	Intergenerational programme	The overarching goal of the intervention was social engagement, with the hopes of fostering intergenerational relationships. Secondary goal: To increase the understanding of dementia among students.	Students visited the care home on a weekly basis where each student was paired with a resident with common interests. Together the pair would work on tasks such as: -Mental stimulation; puzzles, games, reading a book/newspaper -Tactile stimulation; Montessori type activities -Visual arts therapy: drawing, and colouring. At the end of each visit, the students debriefed as a group with the dementia residence staff.	Dementia residence staff, along with student’s teacher.	Half-hour teatime followed by a half-hour of brain-stimulating activities.	Care home	On a weekly basis for 15 weeks. 1-hour long session each.	Students paired with adult with similar interests.
Farina et al. (2020a) England	Educational workshop	This study explored the efficacy and satisfaction of “Dementia Friends”. A dementia education and awareness initiative aimed at reducing stigma using a British adolescent sample.	Adolescents were assigned to either receive Dementia Friends (a 60-min interactive class that teaches about dementia and its effects on people’s lives) or education as usual. All participants completed a series of validated questionnaires pre- and post-intervention, related to dementia attitudes (Brief A-ADS and KIDS).	Volunteer dementia champion and researcher.	Group based interactive tasks with questions and analogies given.	School	Two weeks. First week involves the questionnaire then the week after “Dementia Friends” session. The session lasts 45–60 minutes.	None.
Farina et al. (2020b)	Educational workshop	To evaluate adolescent’s perceptions of the Dementia Friends programme.	Adolescents were assigned to either receive Dementia Friends (a 60-min interactive class that teaches about dementia and its effects on people’s lives) or education as usual. A subgroup of adolescents was placed into 4 focus groups where they were asked questions about the programme one month after its implementation.	Programme was delivered by a Dementia Friends Champion who delivers dementia knowledge to the community and the focus group discussions were delivered by a knowledgeable facilitator.	Face to face group discussions.	School	30 minutes over 1 day.	None.
Liao et al. (2022) Taiwan	Intergenerational programme	To compare the long-term effects of exergaming (Kinect) and companionship programs on attitudes toward dementia and older adults among adolescents.	For the exergaming groups, adolescents led the older participants in bowling, target shooting and Fruit Ninja2 on the Xbox one. Physical exercise was required to complete tasks through visual perception, physical motion, and cooperation. For the companionship groups, adolescents helped perform daily tasks (e.g., card games, singing, painting etc.)	Researcher and care home staff.	Interactive “exergame” console.	Day-care centre	5 weeks & 8 weeks, both same procedures, different durations. Session lasted 40 minutes one day a week.	None.
McNair & Moore (2010)	Intergenerational programme	How would the children’s participation in intergenerational programs impact their attitudes and perceptions towards older adults?	Intergenerational recreation programme. Participants were matched with dementia patients through common interest and personality traits. Six one-hour programs were conducted once per week. The programs involved students’ academic requirements which were completed with the help of the people with dementia.	Researcher and a recreation therapy assistant.	Face to face academic programme, interacting with dementia patients.	Nursing facility	6 weeks 1-hour long programmes performed once a week.	Matched with adults with similar traits in personality and interests.
Parveen et al. (2015) England	Educational workshop	It aims to foster a positive attitude towards those living with dementia and to encourage young people to play an active role in ensuring that they live in a dementia friendly community.	Educational programme completed after initial dementia attitude survey.	Delivered by a knowledgeable facilitator with the help of a teacher.	A talk mixed in with team-based activities.	School	1 hour long educational programme, completed after initial dementia attitude survey. Then another dementia knowledge survey was completed post-programme.	None.
Saif et al. (2020) USA	Educational workshop	Evaluated the effectiveness of various online education strategies concerning Alzheimer’s Disease (AD) risk reduction and brain health in younger populations.	(1) highly interactive webinar lessons narrated by actor Seth Rogen (celebrity webinar), (2) highly interactive webinar lessons narrated by a physician (doctor webinar), (3) minimally interactive video lessons narrated by Seth Rogen (celebrity video), or (4) minimally interactive non celebrity/non doctor narrated video lessons (control).	Session narrated by physician or celebrity.	Lessons via webinar or video; with assignments.	Online educational portal	Not reported.	None.
Song et al. (2019)	Educational workshop	To survey the effect of awareness toward dementia, on Dementia Education Programme (DEP) of adolescents.	Subjects received a Dementia Education Program (DEP) which consist of 9 kinds of education subjects for 4 hours a day, 2 days in total. After the first day, assignments related to dementia were given to be completed.	Not mentioned.	Dementia lessons with assignments.	Online	4 hours a day, 2 days in total.	None.
Wescott & Healy (2011)	Intergenerational programme	To increase positive perceptions of aging and Alzheimer’s Disease in high school students that participated in the Memory Bridge Initiative (MBI) programme.	Students paired with older adult with dementia to share activities and conversation whilst also receiving MBI programme.	Delivered by Maine MBI team members, service-learning teachers and investigator.	Face to face programme with scheduled activities with older adults with dementia.	School, respite care centre and a senior community.	4 week class programme about dementia, communication and empathy including 4 meetings with their “buddy”.	None.

### Description of interventions

There were two intervention types identified out of the ten interventions studied: intergenerational programmes (i.e., contact interventions) which included older adults who are living with dementia, and educational workshops. The ratio of both intervention types was found to be the same in this review.

There were five educational workshops [47,50,51,53,54]. Two studies [47,54] evaluated the Dementia Friends programme. The other three educational workshop interventions [50,51,53] involved the delivery of either a dementia educational programme, a simple class on dementia or using celebrities to deliver dementia knowledge via video.

Five intervention studies were intergenerational programmes [46,48,49,52,55]. Three studies used games, activities, and conversing that involved direct interaction between adolescents and older adults with dementia to be exposed to the practicalities and traits of dementia [46,48,52]. One intervention was interactive but instead of activities both adolescents and older adults with dementia had to help the adolescents with their academic tasks [49]. One intervention encouraged both adolescents and older people to simply be in each other’s presence and to emotionally connect with each other [55].

Eight interventions were conducted in person: three in nursing home centres [46,48,49] and four in school classroom settings [47,50,54,55]. One intervention combined classroom setting, respite care centre and a senior community [52]. Two interventions were delivered solely online [51,53].

In terms of dosage, there were four brief interventions [47,48,50,54]. Both Parveen et al. [50] and Farina et al. [47] implemented a one-hour intervention. Farina et al. [54] was a 30-minute intervention and in Liao et al.’s study [48], total time of the intervention was under four hours. The other interventions ranged between 5 hours and 20 hours [46,48,49,51,55]. Saif et al. [53] was the only intervention that did not state the length of the intervention, which was an educational workshop. Similarly, with Wescott and Healy [52] no time was stated, only the duration of the intervention at a total of four weeks.

### Effectiveness of interventions

Five studies evaluated interventions aiming to improve dementia knowledge as seen in Table 1. All five studies reported an increase in objective or subjective knowledge [46,50,51,53], or a perceived increase in knowledge from qualitative investigation [54]. Chow et al. [46] reported an increase to objective knowledge through an intergenerational programme between a school and a dementia care home. However, the sample size was very small (n = 4), and no control group was involved [46]. Song et al. [51] and Saif et al. [53], both online educational workshops, also found an increase in objective dementia knowledge post-intervention. Saif et al. [53] is the only randomised controlled trial and reported all four intervention groups to show an increase in knowledge of risk reduction in Alzheimer’s post intervention, most notably through the celebrity video and celebrity webinar [53]. Parveen et al. [50] found adolescents to report an increase in subjective knowledge through their ‘Dementia Detectives’ educational workshop.

Farina et al. [54] in their qualitative investigation of the ‘Dementia Friends’ educational workshop explored subjective knowledge and found that adolescents perceived an improvement in their knowledge of dementia. Four out of four education focused interventions found an increase in knowledge of dementia [50,51,53,54]. The sole intergenerational programme that measured dementia knowledge also found an increase [46].

Eight studies evaluated interventions aiming to improve attitudes towards dementia [46–49,51,52,54,55]. Four out of five intergenerational focused interventions reported an improvement in attitudes [46,48,52,55]. The remaining intergenerational intervention found no improvement in attitudes towards dementia [49]. Although McNair and Moore [49] did not find any significant changes to attitude, they found an increase in confidence in ability to help individuals with dementia.

Three out of three education-focused interventions reported an increase in attitudes [47,51] or a perceived increase in attitudes from the qualitative investigation study [54]. Farina et al. [47] used an interactive educational format and found an increase to attitudes in the KIDS questionnaire [58] and no significant change in attitudes pre and post intervention in the brief A-ADS [56]. However, the KIDS questionnaire [58] was originally developed for a younger (age 9–12) Australian sample, which may impact on confidence in this finding [47]. Farina et al. [47] had significantly more participants (n* *= 301) taking part in the intervention compared to Farina et al. [54] (n* *= 30) and Song et al. [51] (n = 32). However, even with the smaller sample size of Farina et al. [54], through their qualitative analysis of the implementation of an interactive educational intervention also found adolescents attitudes to increase. Similarly, the smaller sample size of Song et al. [51] which implemented an educational intervention online found an increase in adolescent attitudes and awareness towards dementia.

## Discussion

This review is the first to chart and synthesise what is currently known about adolescent knowledge and attitudes towards dementia and how this can be enhanced through intervention. This review utilized the JBI methodology for scoping reviews and identified 21 publications, reporting 16 studies published between 2005 and 2022. The small number of studies identified in this scoping review report important gaps in dementia knowledge among adolescents. Many adolescents reported misunderstandings about dementia treatment, the trajectory and chronicity of the disease. Conversely, attitudes towards dementia were largely reported to be neutral-positive. However, adolescents living with a relative with dementia often held predominantly negative attitudes towards the neurocognitive condition. Intervention research in this area is sparse, with a high degree of heterogeneity and only one randomised controlled trial.

Adolescent objective knowledge of dementia was reported as low across a number of studies. As may be expected, this is consistent with the general population, where a systematic review [59] of international population-level surveys reported a low level of objective knowledge among adults. For example, the common misconception that dementia is a normal part of ageing and is not preventable [59]. This systematic review indicated that public understanding of dementia may be improving over time. With a smaller evidence-base in adolescents specifically it would be difficult to draw similar conclusions. However, exposure to a family member with dementia appears to be a common predictor of heightened knowledge in both the adolescent and adult populations and with increasing prevalence of dementia [4], adolescent knowledge could also be improving through this mechanism. Importantly however, misconceptions exist even among adolescents with a family member of dementia. For example, in Fuh et al. [36], a third of adolescents believed that dementia is a normal part of the ageing process. There is an opportunity to strengthen downstream population level understanding of dementia by targeting adolescents, who appear to have the same desire to increase their dementia knowledge as can be found in adult populations [e.g., 7].

Adolescent attitudes towards dementia were largely reported as neutral to positive across studies included in the review, though with a significant subgroup of individuals reporting negative attitudes. As with research on adolescent knowledge, this was based on a small number of articles (n = 5), some of whom did not use the validated assessment tools now available to assess key public health constructs within dementia research [60]. The included studies reported male gender and having a family member with dementia as predictors of more negative attitudes towards dementia [14,40], suggesting similar targets for intervention as with the adult population [61]. A more in-depth and theoretically informed understanding of predictors of attitudes towards dementia is needed however, assessing a broader range of predictors and those which are potentially modifiable. Longitudinal research is also a significant gap in this field across the life course [62], which would be informative in terms of understanding how dementia-related stigma develops across time. This is important given that several theories [e.g., *Impressionable Year’s Hypothesis*; 63] have suggested that adolescence is a key time for attitude change, where experiences can have a more significant impact on a young person’s beliefs and where there is still a degree of flexibility for attitude change which decreases into adulthood.

Very few studies gathered information on how stakeholder’s perceptions of adolescent knowledge and attitudes can be enhanced. To gain a further understanding of this, using and developing interventions with a co-design approach with young people may gather increased input around stakeholder perceptions in enhancing these concepts [64].

The scoping review identified a small number of studies (n = 10) evaluating interventions to improve dementia knowledge and attitudes in adolescents, with largely quasi-experimental designs, two qualitative studies and one randomised controlled trial. The small body of research in this area is reflective of the evidence-base on interventions to reduce dementia-related stigma in the general population [65]. Intervention formats included in the scoping review aligned to the first two categories of Corrigan and Penn’s stigma reduction framework [66]; education and contact (to provide interaction with people with dementia). This framework identifies how stigma operates through stereotypes, prejudice and discrimination, highlighting the impact of dementia stigma in adolescents and supporting intervention design [66]. All the studies included in the review aimed to increase either dementia knowledge or attitudes. All four studies assessing change in knowledge pre- and post-intervention reported an increase in knowledge among adolescents. Additionally, a qualitative study [54] reported adolescents to perceive a change in their knowledge from taking part in the intervention. Six out of the seven studies assessing change in attitude pre- and post-intervention, reported improved attitudes [46–48,51,52,54,55]. Additionally, a qualitative study [54] reported adolescents to perceive a change in their attitudes from taking part in the intervention. Another qualitative study found their attitudes to have improved after the intervention [55]. Five of these studies evaluated intergenerational programmes [46,48,49,52,55] and five studies evaluated educational workshops [47,50,51,53,54]. Educational and intergenerational interventions developed to date appear similarly effective in improving adolescent knowledge and attitudes. However, there was only one randomised controlled trial identified and all the intervention studies would therefore likely include a high risk of bias. This is an underdeveloped evidence base, with the need for more high-quality trials. The broader body of research however evidences that intergenerational interventions influence attitudes towards dementia in healthcare professionals and young adults [67] adding further reassurance that this is a useful intervention strategy for the adolescent population.

The scoping review has several strengths. We used a rigorous JBI methodological approach which ensured the scoping review was conducted to a high standard. Through conducting further reference and citation harvesting, a number of relevant studies were included that were not found initially through the four original database searches. Although there is a dearth of research in this area, the included studies provide insight into the knowledge and attitudes of adolescents towards dementia and interventions which suggest promise in improving outcomes. There are also several limitations which should be discussed. The scoping review was limited to English language publications, and this may have contributed to the majority of included studies being limited to high-income-countries. The lack of evidence-base in low-and-middle-income countries means findings are not representative of the global adolescent population. There is suggestion that culture and social context can influence dementia-related attitudes in adults [68], and so this is likely to also be the case with adolescents.

### Recommendations for future research

Of the countries monitored by the WHO via the Global Dementia Observatory [4], 36/43 countries with dementia action plans include objectives to address dementia awareness, stigma reduction, and encouragement of dementia-friendly communities. The majority of research relating to dementia awareness and stigma reduction relates to the adult population [65]. In order to implement a public health approach, which is focused on improving the readiness of all groups within the general population, we urgently need to increase our understanding of dementia knowledge and attitudes among adolescents. This scoping review has identified that this is a small and underdeveloped research area, with an aligned dearth of high-quality interventions. As a priority, there is a need for more rigorous research to understand the prevalence and risk factors of poor dementia-related knowledge and negative attitudes. Research is needed which uses the validated assessment tools now available [56,69] and which is theoretically informed, ideally with a focus on identifying risk factors which may be modifiable via public health intervention. The majority of the current research utilises convenience samples with adolescents recruited from secondary schools. Research which is conducted with samples more representative of the general population would add value, alongside longitudinal research to understand if and how negative attitudes towards dementia develop. Finally, there was a dearth of high-quality evaluations of interventions identified in the scoping review. Further intervention research is needed, informed by evidence and theory. The conduct of feasibility trials would help advance the quality of the science in this area towards conducting future randomised controlled trials. The limited size and quality of the evidence-base precludes strong conclusions on implications for practice. However, the need to target adolescent knowledge of dementia, over attitudes, appears most pertinent, and use of intergenerational or educational intervention strategies.

## Conclusion

Adolescents’ objective knowledge of dementia is reasonably low, with evidence that various misconceptions about dementia are prevalent. Although attitudes towards dementia are largely neutral to positive, there is a sizeable subgroup suggested to report more negative attitudes. We currently have very little understanding of how dementia-related stigma may develop across adolescence and early adulthood. Interventions to date which include an intergenerational programme and educational workshop are suggested to be similarly effective at increasing knowledge and improving attitudes towards dementia. However, this is a small research area, with significant potential for development within larger efforts to implement a public health approach to dementia.

## Supporting information

S1 Appendix Preferred reporting items for systematic reviews and meta-analyses extension for scoping reviews (PRISMA-ScR) checklist. (DOCX)

S2 Appendix Search terms. (DOCX)

S3 Appendix JBI extraction form. (DOCX)
