# Supplementary material for: What is known from the existing literature about adolescent knowledge and attitudes towards dementia and interventions to enhance this? A scoping review
Source: PLoS One. 2025 Sep 8;20(9):e0322423. doi: 10.1371/journal.pone.0322423 (PMC12416682; doi:10.1371/journal.pone.0322423)
Supplement: S2 Appendix — (DOCX) [file pone.0322423.s002.docx]

**CINAHL**

S1 (MM "Dementia")

S2 dementia OR alzheimer*

S3 (MM "Adolescent Psychology") OR (MH "Adolescent Health") OR (MM "Adolescent Development") OR (MM "Adolescence") OR (MM "Adolescent Behavior")

S4 adolescen* OR teen* OR juvenile* OR youth* OR "young carer" OR "young person" OR "young people"

S5 (MM "Knowledge") OR (MM "Knowledge Bases") OR (MM "Health Knowledge")

S6 knowledge OR attitude* OR comprehen* OR perception* OR belief* OR understanding OR insight* OR view* OR approach* OR acknowledge* OR awareness OR recognise* OR recognize* OR know

S7 S1 OR S2

S8 S3 OR S4

S9 S5 OR S6

S10 S7 AND S8 AND S9

S11 Limiters - Publication Year: 2000-2022; English Language; Human
Expanders - Apply equivalent subjects
Search modes - Boolean/Phrase

**Embase**

1 Dementia

2 (dementia or alzheimer*)

3 adolescent development* or adolescent behavior* or *adolescent* or adolescent health

4 (adolescen* or "young carer" or juvenile* or teen* or youth* or "young person" or "young people")

5 knowledge discovery* or knowledge* or knowledge base*

6 (knowledge or attitude* or belief* or view* or insight* or know* or recognise* or recognize* or comprehen* or approach* or awareness or understanding or perception* or acknowledge*)

7 1 or 2

8 3 or 4

9 5 or 6

10 7 and 8 and 9

11 limit 10 to (human and english language and exclude medline journals and last 24 years)

**MEDLINE**

1 Dementia

2 (dementia or alzheimer*)

3 Psychology Adolescent* or Adolescent Behavior* or Adolescent* or Adolescent Development*

4 (adolescen* or "young carer" or juvenile* or teen* or youth* or "young people" or "young person")

5 Knowledge* or Health Knowledge, Attitudes, Practice* or Knowledge Discovery* or Knowledge Bases* or "Knowledge of Results, Psychological"

6 (knowledge or attitude* or perception* or understanding or comprehen* or awareness or belief* or insight* or view* or approach* or acknowledge* or recognise* or recognize* or know*)

7 1 or 2

8 3 or 4

9 5 or 6

10 7 and 8 and 9

11 limit 10 to (english language and humans and last 24 years)

**PsycINFO**

1 Vascular Dementia* or exp Dementia* or Dementia with Lewy Bodies* or Senile Dementia*

2 (dementia or alzheimer*)

3 Adolescent Behavior* or Adolescent Attitudes* or Adolescent Development* or Adolescent Psychology* or Adolescent Health*

4 (adolescen* or "young carer" or teen* or juvenile* or "young person" or "young people" or youth*)

5 1 or 2

6 3 or 4

7 5 and 6

8 limit 7 to (human and english language and last 24 years)
