# Supplementary material for: What is known from the existing literature about adolescent knowledge and attitudes towards dementia and interventions to enhance this? A scoping review
Source: PLoS One. 2025 Sep 8;20(9):e0322423. doi: 10.1371/journal.pone.0322423 (PMC12416682; doi:10.1371/journal.pone.0322423)
Supplement: S3 Appendix — (DOCX) [file pone.0322423.s003.docx]

JBI Extraction Form

| Author(s) & Year |  |
| --- | --- |

| Location (school, community or other) |  |
| --- | --- |

| Name & Date Completed |  |
| --- | --- |

| Research Design (e.g. cross-sectional, qualitative, quantitative etc.) |  |
| --- | --- |

| Population Type (setting recruited from e.g. students/young carers/youth club) |  |
| --- | --- |

| Gender (male, female, other, or not displayed). |  |
| --- | --- |

| Age Range |  |
| --- | --- |

| Inclusion criteria |  |
| --- | --- |

| Aims |  |
| --- | --- |

| Methodology (e.g. qualitative – semi-structured interviews using IPA methodology or quantitative using MANOVA etc.) |  |
| --- | --- |

| Intervention(s) (yes/no. If yes include type of intervention, effect (size), length of intervention that was implemented, control groups etc) |  |
| --- | --- |

| Results (quantitative - effect size, statistical figures. Qualitative – themes) |  |
| --- | --- |

| Limitations |  |
| --- | --- |

| Recommendations for future research |  |
| --- | --- |

| Conclusions |  |
| --- | --- |
